# Falling through the gaps: exploring the role of integrated commissioning in improving transition from children’s to adults' services for young people with long-term health conditions in England

**DOI:** 10.1177/1355819617752744

**Published:** 2018-02-23

**Authors:** Gregory Maniatopoulos, Ann Le Couteur, Luke Vale, Allan Colver

**Affiliations:** 1 5994 Senior Research Associate, Institute of Health and Society, Newcastle University, UK; 2 5994 Professor of Child & Adolescent Psychiatry, Institute of Health and Society, Newcastle University, UK; 3 5994 Health Foundation Chair in Health Economics, Institute of Health and Society, Newcastle University, UK; 4 5994 Emeritus Professor Community Child Health, Institute of Health and Society, Newcastle University, UK

**Keywords:** integrated commissioning, transition, United Kingdom, young people with long-term conditions

## Abstract

**Objectives:**

To explore the role of integrated commissioning in improving the transition of young people with long-term conditions from child to adult services. We aimed to identify organizational and policy gaps around transition services and provide recommendations for integrated commissioning practice.

**Methods:**

Semi-structured in-depth interviews were conducted with two groups of participants: (1) twenty-four stakeholders involved in the commissioning and provision of transition services for young people with long-term conditions in two regions in England; (2) five professionals with national roles in relation to planning for transition. Transcripts were interrogated using thematic analysis.

**Results:**

There is little evidence of integrated commissioning for transitional care for young people with long-term conditions. Commissioners perceive there to be a lack of national and local policy to guide integrated commissioning for transitional care; and limited resources for transition. Furthermore, commissioning organizations responsible for transition have different cultures, funding arrangements and related practices which make inter- and intra-agency co-ordination and cross-boundary continuity of care difficult to achieve.

**Conclusions:**

Integrated commissioning may be an effective way to achieve successful transitional care for young people with long-term health conditions. However, this innovative relational approach to commissioning requires a national steer together with recognition of common values and joint ownership between relevant stakeholders.

## Introduction

Access to health care for young people with long-term conditions as they progress from child to adult health services should be straightforward. However, the health, social and educational outcomes for these young people in adulthood are often poor and are not as good as those for people without long-term conditions.^1–3^ One risk factor likely to be contributing to these poor outcomes is unsatisfactory health care transition.^4^

Health care transition is the ‘purposeful, planned movement of adolescents and young adults with chronic physical and medical conditions from child-centred to adult-oriented health care systems with the goal of providing health care that is uninterrupted, co-ordinated, developmentally appropriate, psychologically sound, and comprehensive’.^5^ It is a multi-disciplinary and holistic process which addresses the medical, psychosocial, educational and vocational needs of young people with long-term conditions.

Commissioning is the process by which public services are planned, contracted and monitored to meet population needs. Commissioning in the United Kingdom (UK) involves much more than procuring services and managing transactional issues related to the acquisition of health care services; it ranges from the health needs assessment for a population, through the clinically based design of patient pathways, to service specification and contract negotiation or procurement, with continuous quality assessment.^6^ Most of the National Health Service (NHS) commissioning budget is now managed by clinical commissioning groups (CCGs). These are groups of primary care practices which come together for a defined geographical area to commission the best services for their patients and population. At a national level, NHS England commissions specialized services, primary care and some public health services.

Recent UK policy and best practice guidance documents suggest that an integrated approach to commissioning for transition could improve the experience of the patient and family.^4^ In this context, integrated commissioning can be broadly defined as the process whereby joint institutional arrangements, within health and between health and social care settings, guide the organization, funding and provision of services. This can take the form of a ‘partnership, alliance or other collaboration’ between professionals and organizations working together at all stages of the commissioning process; from the assessment of needs, to the planning and funding of services, and the monitoring of outcomes.^7^

Such integrated commissioning should facilitate the delivery of person-centred care, i.e. placing individual needs at the centre of the healthcare practice.^8–11^ Conversely, lack of integrated commissioning may prevent the delivery of person-centred care during transition, which in turn increases the risk of negative patient experiences of health care and poor health and social outcomes following transition.^4,12^ In the UK in 2010, a review of children's and young people’s services emphasized the importance of integrated commissioning for transition, urging that ‘arrangements must be agreed, regarding funding and other matters, to address the changing needs of children and young people as they mature, including greater continuity of care into adulthood. Ensuring a smooth transition between child and adult services should be a priority for local commissioners’.^13^

In recent years, health and social care commissioning in the UK has been subject to a number of fundamental reforms, aimed at improving the quality of health and social care services^14–15^ by stimulating a more strategic and integrated approach to the planning and use of resources.^8^ Despite the UK government’s policy aspiration for integrated commissioning, in practice, progress has been slow and patchy.^8,16,17^ Commissioning of health and social care services is often disjointed and fragmented.^8,18^ Commissioning transition from child to adult services for young people with long-term conditions is a particular challenge because of the complexity of the types of health care required, the developmental needs of each individual and family, and the multi-disciplinary, inter-agency nature of the service provision.

Against this background, this paper explores the role of integrated commissioning in improving the transitional care of young people with long-term conditions. The paper sets out the experiences of a number of key stakeholders involved in commissioning and provision of transition services. By understanding these particular examples of transition services, we aim to identify organizational and policy procedures in relation to transitional care to inform recommendations for improved integrated commissioning practice. We draw upon one strand of a five-year research Programme (the National Institute of Health Research Programme on Transition). The overall programme aimed to cost-effectively improve the quality of life of young people with long-term health conditions (http://research.ncl.ac.uk/transition/). The aim of the commissioning strand was to identify potential facilitators and barriers to successful commissioning for transition; that is, to identify what constitutes successful commissioning for transition, and to identify the challenges of commissioning for transition. Here, we report a secondary analysis of our data on commissioning more generally to examine the specifics of integrated commissioning.

## Methods

Semi-structured in-depth interviews (29 in total; see Table 1) were conducted between April and August 2014. Participants were purposively selected according to their role and involvement in transitional care, from both a commissioning and service delivery perspective, in two geographical regions in England, on the recommendation of members of a research external advisory steering group. Both regions were engaged in the implementation of services that included specific provision for transitional care (from child to adult services) of young people with long-term conditions. Potential interviewees were sent an email invitation which briefly outlined the aims and objectives of the study. Those agreeing to participate were also invited to recommend additional candidates for interview. Individuals who agreed to participate in the study were provided with information sheets in advance, and once any questions were answered, participants gave informed consent prior to the start of the interview. All interviews were digitally recorded, anonymized and transcribed in full. Interviews were typically around 60 min in length and conducted on an individual, face-to-face basis.

**Table 1.** List of interviewees.

Organization	Number of interviews	Interviewees	Region
Health and social care commissioning organization	5	Commissioners/managers	Region 1
NHS	1	Senior manager	Region 1
NHS	1	Senior clinician	Region 1
NHS	2	GPs	Region 1
Local Authority	2	Transition planning workforce	Region 1
Voluntary sector	1	Regional coordinator	Region 1
Health and social care commissioning organization	8	Commissioners/managers	Region 2
NHS	1	Senior clinician	Region 2
NHS	1	GP	Region 2
Local Authority	2	Transition managers and co-ordinators	Region 2
NHS	1	Senior manager	National
NHS	2	Clinical lead	National
Voluntary sector	2	Transition leads	National
Total	29		

The final sample included: (1) twenty four individuals involved in the commissioning and provision of transition services for young people with long-term conditions; (2) five individuals with national roles as ‘clinical champions’ in relation to transition planning. Participants included (i) senior clinicians, NHS clinical leaders and directors, commissioners and health care managers across CCGs, Health and Wellbeing Boards, and Local Authorities with roles relevant to transition planning and service co-ordination; (ii) local general practitioners (GPs); and (iii) the voluntary sector. Many interviewees had multiple roles both as commissioners and providers of transition services and therefore their voices often represented their experiences from both areas of practice.

## Data analysis

We undertook an inductive thematic analysis^19^ which focused on the role of integrated commissioning in improving transitional care for young people with long-term conditions. Emerging themes were identified for each participant and then compared within and across the interviews. One member of the research team (GM) undertook the analysis of the interview data. These were then reviewed and discussed at wider research team meetings, with any discrepancies resolved through this process.

## Ethics

A positive ethical opinion was obtained from Newcastle University Faculty of Medical Sciences Ethics Committee (ref: 00767/2014).

## Results

Analysis of the data generated four broad themes relating to challenges surrounding integrated commissioning for transitional care for young people with long-term conditions: (1) national and local policy to guide integrated commissioning; (2) integrated commissioning for transition within health and between health and social care settings (i.e. an absence of joint institutional arrangements); (3) institutional separation of health and social care services, roles and responsibilities; and (4) communication and co-ordination.

### (1) National and local policy on transition to guide integrated commissioning

Participants acknowledged the important role of integrated commissioning to facilitate transitional care.

I think if you look at the political direction, it’s all about integration and you know closer working with councils. But that’s all, obviously, political…a sort of political decision isn’t it really? But there could be some merits in that, I mean, you know, would make a lot of sense. There’s so much joint working now and also so many issues about, kind of, the divide and the funding … (Commissioner/manager 4, Region 1)

However, none of the participants identified transition as an NHS priority, and this ultimately translated to resource limitations for transition. For some participants, the lack of a national policy on transitional care was partly attributed to competing priorities and targets set by government for different services; and further attributed to the limitations of different funding streams which could hinder meeting young people’s needs in a holistic way.

I think that’s probably one of the barriers in that actually does anybody really give it, does it get a priority? Is it, you know, the fact that I’ve had to scout around trying to find out what we do and, and the fact that talking to people about transition, they say, “Oh yeah, yeah, yeah. We’re doing this on diabetes” … and I think, “Oh right, okay.” But I’ve, I’ve had to go and find out … No, so it doesn’t have a profile … (Commissioner/manager 6, Region 2)

I think things like transition, whilst we’re aware that it’s an issue, we’re also acutely aware that there are bigger issues at stake here, like, the affordability of NHS services to meet people’s needs in [the local area], and the country, for the future … So you tend to find that the big issues, like the fact that we’re about, potentially, about £8 million short in terms of budget this year is much more of a priority than transition, clearly. So I think things like transition, you know, suffer as a result … (Commissioner/manager 5, Region 1)

Participants also reported that funding arrangements were fragmented between and within different health and other local care settings and guidance in relation to use of funding was not always clear.

I think one of the things I’ve talked a lot about is the structural barriers built in by legislation and how the local authority and the CCGs divide up money. I think they are huge barriers. I think the things that get the goat of families and parents is when you’ve got a process, you’re told what the process is, it seems sensible and it doesn’t work … (Commissioner/manager 3, Region 2)

Some participants reported that as well as no formal local policy to guide integrated commissioning on transitional care, there was no national guidance to frame commissioning responsibilities between and within care settings.

In terms of sorting out the, the funding, the split of funding there’s no national guidance, no national policies … Everywhere has different splits, have different ways of doing it. It’s notoriously hard thing … is it about health need or social need, if you’re continuing a health care and looking at shared care funding and all, all sorts of things. There’s endless, I think, debate and angst at, about how that sort of, how that cuts between the agencies … (Commissioner/manager 4, Region 1)

Given the absence of a structured commissioning approach, it was suggested that specific guidance on transitional care was required to enable integrated commissioning decisions about funding the provision of transitional care.

I think commissioners don’t get it, they don’t understand transition. You’re either a child or an adult in the eyes of the commissioners and they really don’t understand what they’re commissioning … it’s small for them; they’ve got other priorities … I would love to see some type of commissioning guidance or something that we could take along to different commissioners … as much statutory guidance as possible … (Voluntary sector leader 1, National)

As a consequence of lack of guidance and resources to support transitional care planning and commissioning across care settings, one participant emphasized the need for greater transparency with young people and their families about the availability (or lack) of resources for transitional care.

We’ve got to be more transparent about the resources … So our aims are to always try and be clear, as clear as possible with families and as transparent about the resources that are available for … I don’t think we’ve been very honest with young adults and their families about the amount of resources that are available. I think we’ve just kept that very quiet … What we’re definitely trying within children’s services is to say, “This is your fair share of the resources that we have available.” It’s changing the culture of the relationship that we had … (Transition planning workforce 2, Region 1)

### (2) Integrated commissioning for transition between child and adult services both within health and between health and social care settings (i.e. an absence of joint institutional arrangements)

A number of participants reported that there was a lack of clarity on integrated pathways for the effective commissioning for transition. In particular, participants reflected on issues related to the strict use of age as a criterion for accessing different services which could also contribute to poor transition. In this context, some participants criticized the assumption that chronological age alone indicates a readiness for transfer from one service to another, thus disregarding/ignoring the different developmental needs of young people and the complexity of adolescent development.

But if we’re talking about moving into adulthood then health care is particularly complex … the criteria for service, is the biggest reason for complaint. You know, because, “My child’s needs hasn’t [sic] changed ‘cause they had a birthday, yet what you are willing to support me with has changed. Why is that?” And where government is saying, “Well we need to go through to 25” that’s fine, but as long as there’s a, an 18-year-old cut off and there isn’t the funding there to support that, the world isn’t going to change … (Commissioner/manager 2, Region 1)

Participants reported that these different age cut-offs within health and between sectors such as health and education could result in poor transitional care pathways, increasing the risk that young people might be excluded from particular services that they could potentially benefit from. For example, in the context of mental health services, there is still considerable variation in the age chosen for discharge from child services and access to adult services. These discrepancies affect commissioning arrangements for transitional care.

We’ve had an issue highlighted this week around a 17-year-old who’s not known to services and is struggling to get into services because children’s services say “oh we stop at 16” and adult services say “we don’t start until 18”. Because she’s not in a service it proves difficult so we know we’ve got a lot to do still … (Commissioner/manager 3, Region 2)

Participants suggested that a lack of clarity on service availability and the operation of different eligibility criteria between child and adult services could become a major barrier towards integrated commissioning for transitions. Examples of these discrepancies and the consequences for access to service were described by both physical and mental health services.

We have got around the contracts that when somebody turns 18 they might use a respite service but it’s only registered up until the point when they’re 18 and once they hit their 18th birthday they’re no longer able to use that service. The children’s respite services are commissioned one way and they’re different … [the adult services] don’t have the capacity to provide that service in the same way, and that can be about them not having the physical space and the beds or that the person might not actually want to or be able to take up that resource … we’ve got quite a lot of difficulty trying to sustain a care package that might have been working quite well but has needed to change when they’re 18 … (Transition manager 1, Region 2)

A view consistently expressed by many participants was that adult services generally have higher thresholds for accessing services, and typically services reduce as young people move from child to adult services.

Almost half the kids who reach the transition boundary for CAMHS [Child and adolescent mental health services] simply drop through the care gap. Their care ceases. Some of them appropriately because they don’t need adult care, but a lot of them despite having clinical needs, they just simply drop through the care net … (NHS clinical leader 1, National)

Families will tell you that they’ve got a really good wrapped around, cosy service while their children are young, when they hit 18 eligibility changes … (Commissioner/manager 1, Region 1)

### (3) Institutional separation, roles and responsibilities

Despite the UK government’s policy aspirations for integrated commissioning, participants thought that the recent reforms to the ‘operating framework’ of the NHS across the health and social care interface^20,21^ present a number of challenges to the integrated commissioning of care pathways for transition services.

It’s been difficult in the last year since all of the changes … previously when it was the PCTs [primary care trusts] and our specialised commission team sat within a PCT so and worked on behalf of PCTs so it was easy we would know who to go to, who to speak to … it’s very difficult now with CCGs … a lot of people have changed … it’s also difficult to try and get people to take responsibility for certain areas I think there’s a bit of to-ing and fro-ing between NHS England and CCGs they’ll say you know that’s specialised commission responsibility we’ll say we think it’s CCG responsibility … (Commissioner/manager 5, Region 1)

Well it, I mean it definitely impacts commissioning because, as I say, at the moment it’s unclear as to how much, you know, what the CCG are paying for… so it’s then difficult to say, “Well actually the CCG shouldn’t pay for that, they, you know, the local authority should pay for that part of this service.” And it’s also difficult to unpick how much the foundation trust itself is subsidising the service … (Commissioner/manager 3, Region 2)

Although the new commissioning landscape emphasizes the important role of joined-up working between and within health and social care commissioners,^8^ for some participants the reforms appeared to hinder the shared value base and purpose underpinning commissioning practice for transition.

I think it is so complicated and we, it’s so multi-agency isn’t it really? You know, and we don’t have a shared value base of what we’re trying to achieve with young people and their families … (Transition planning workforce 2, Region 1)

Some participants raised concerns about the institutional separation and cultural differences between health and social care commissioning as well as between child and adult services and the implications for integrated commissioning for transition.

So for me there’s a difference about what commissioning can do, what commissioning can’t do … What government and CCGs and local authorities could do … and I can see some solutions to all of those, the one I can’t see a solution to is the fact that legally we deal with children and young people differently from adults, and therefore the health services are structured differently for children as they are to adults … (Commissioner/manager 1, Region 1)

I think again it feels very much like a capacity issue in terms of them feeling like it’s another thing to do above and beyond what they’ve been used to doing … I think everybody is feeling the stretch at the moment. I know we work with two main acute trusts and certainly at one of them we just couldn’t get the adult provisory, been having meetings around transition and we had everybody from children’s services that we needed around the table but we really struggled to get the adults on board and I think that’s a lot, I don’t think the will isn’t there … I think … it’s partly coming from management that they don’t, that they see transition as a children’s service problem not necessarily an adult’s one. (Commissioner/manager 2, Region 2)

Although the value of co-location and inter-professional working were emphasized by some participants, an important persisting problem was that services for children and adults are commissioned separately and use a different organizational structure.

Adult services are structured completely different to children’s services … in children’s services health are much more likely to provide a coordinated approach to your care. In adult services you are expected to provide that coordination of your care … (Commissioner/manager 1, Region 1)

So everyone’s in a bit of the dark and the other side of that is children services don’t talk to adult services, they’re two very different systems, individual budgets, direct payments are running in a different way in children’s to the way they are in adults, there’s not as much freedom, it’s very specific, you can do this, this, this, whereas in adults you’ve got more freedom … (Voluntary sector leader 2, National)

In this context, structural barriers across health, education and social care can result in poor engagement, discontinuity of care and commissioners feeling unclear about their role and responsibilities in the process of commissioning for transitional care.

The other thing the government do is they’ll send guidance on what they think a health need is and what an education need is, or a social care need is, which again creates barriers. So, for instance, if you are peg fed when you’re at home you could say that’s a health care need because you need to be fed to live. While you’re at school, school are responsible for making sure you can access education, you can’t access education if you’re hungry, so it’s, is it then their responsibility to feed you? (Commissioner/manager 4, Region 1)

### (4) Communication and co-ordination between commissioning agencies

The institutional separation and the absence of a co-ordinated approach to providing services across care settings can result in poor inter- and intra-agency communication which in turn was seen as a barrier hindering commissioning for transition.

I think the biggest barrier is going to be the lack of communication probably between commissioners … it’s a little bit of a us versus them situation at the moment in terms of who’s got the money for what and that’s probably going to be the biggest barrier … (Commissioner/manager 5, Region 1)

Sadly within the hospital there is a bit of a division between the adults and children’s, there’s no overlap, there’s not an adolescent unit or anything like that [Okay], which is very disappointing … (Senior Clinician, Region 2)

This was particularly the case when there is a need to develop and commission joined-up packages of care and to identify integrated funding solutions (pooled budgets) that go beyond the remit of child services.

I think where we fall down is that some of the high care needs panels do not include adult colleagues for commissioning … I agree funding up to the age of 18, somebody else needs to pick up the ball past 18. And in order for that to happen, certain things need to happen beforehand … So the challenge for us as commissioners is to get adult commissioners to note the fact that those children need assessment before they turn 18 … (Commissioner/manager 1, Region 1)

Lack of engagement for joint planning was perceived to be a major barrier to integrated commissioning not only within health but also in social care too.

One of the problems we’ve had with our team with continuing care itself is a lack of social work input … children or young people that need a decisions support tool filling in … for adults actually you have to have a social worker at that meeting … (Senior Clinician, Region 2)

These limited opportunities were perceived to be due, at least in part, to a lack of capacity and senior management commitment to support joined up commissioning for transition.

It feels very much like a capacity issue in terms of them feeling like it’s another thing to do above and beyond what they’ve been used to doing … we work with two main acute trusts and certainly at one of them we just couldn’t get the adult provisory, we’d been having meetings around transition and we had everybody from children’s services that we needed around the table but we really struggled to get the adults on board and … I think it’s partly coming from management that they don’t, that they see transition as a children’s service problem … (GP, Region 2)

A failure to share information between different agencies and sectors was also identified as a key factor contributing to poor co-ordination of integrated commissioning for transition.

The transfer of information from one professional to another, despite me thinking, “That is the easiest and simplest thing to do” it’s not always the case. And you might transfer the information electronically or make it available, [but] whether that person reads that … (Commissioner/manager 4, Region 1)

Participants proposed that challenges to co-ordinating commissioning arrangements for transitional care were compounded by perceptions about professional roles, responsibilities and competencies.

Medical models that say, “I am a trained paediatrician and therefore once you’re 18 I don’t understand anything about you.” Or, “I am not a paediatrician and therefore I can’t talk to you ‘til you’re over 18 ‘cause I don’t understand anything about you.”… I think, so the medical model constructs around children’s people and adult people, professionals. I think added to that is, as I say, the law … (Commissioner/manager 1, Region 1)

In addressing these issues, some participants suggested that developing and maintaining good relationships between commissioners and providers is central to integrated commissioning for transition.

For me it’s about collaboration, proper collaboration where the commissioners, the providers … and the family … are proper partners in the whole process. I don’t see that happening, where they’re all brought in to the same ideal scenario … through that process there’s a feeling of trust in the fact that those people, that little huddle of folk could make sure that they do the right thing … (Regional coordinator, Voluntary sector, Region 1)

## Discussion

This inquiry raises important questions about the current state of integrated commissioning for transitional care for young people with long-term conditions. The majority of participants acknowledged the relevance of integrated commissioning but perceived a lack of national and local policy for integrated commissioning to guide or change local service practices. Further, there was a perception that integrated commissioning did not consider the reality of resource constraints in different services. Differences between commissioning organizations in terms of cultures, funding arrangements and related practices raised the concerns of most participants about the adverse impact this usually had on inter- and intra-agency co-ordination and cross-boundary continuity of care. There was no sense that such diversity could facilitate innovative integrated commissioning practice. For the interviewees, the two biggest barriers to effective commissioning were (i) lack of communication between commissioning agencies; and (ii) failure to develop jointly agreed institutional arrangements both within health and between health and social care settings.^22–25^ This is consistent with previous studies which have shown how the fragmented nature of NHS and social care services and the differences in organizational cultures, values and working practices hinder joined-up working and cross-boundary continuity of care.^26–29^

Despite a satisfactory number of participants in the study, a potential limitation is that the majority of participants were drawn from just two geographical regions in the UK; this might influence generalizability across different health systems. However, our findings are consistent with similar issues identified in a recently published systematic review focussing on integration between care settings.^30^

The apparent absence of integrated commissioning for transition identified in this study poses a number of challenges for the delivery of integrated care. For example, for some young people with particular long-term conditions, there was no commissioned designated service for the young person to be transferred to.^12^ Participants discussed how young people and their families often become worried as the young person moved towards the time when they leave children’s services, and the difficulty of living with the uncertainty of what support they will receive in future. Even where there are services to transfer to, participants thought that young people and their families found the uncertainty as being like a ‘cliff edge’. Families move from a firm ground where there might be a co-ordinated care plan involving different services that know the child to the ‘unknown’ where the adult more symptom-orientated services expect the service user to self-manage their condition and co-ordinate their own care, even when they have multiple needs.^4^ This concern about a ‘cliff edge’ in care provision may be accompanied by further concern about limited expertise in the adult services especially when the long-term condition is rare. In this situation, concerns are that the available provision might not focus on the relevant needs of young people and their families. Sometimes these difficulties may result in the child services continuing to care for the young person beyond the usual age boundaries in an attempt to avoid the young person falling into the gap.^26^ However, this in turn means that adult services are unable to gain the working knowledge about how to provide developmentally appropriate care for this particular client group.

The need to reconfigure the relationship within health and between health and social care commissioning to support shared responsibility and ownership for transitional care of young people is relevant whatever the local model of health and social care provision.^31^ This requires policy-makers, commissioners and service providers to work together. Taking a joint and shared responsibility within health and between health and social care commissioning is a necessary step for the design of integrated commissioning to deliver effective care to young people with long-term health care needs and their families. Several of those interviewed emphasized the benefits of shared and joint responsibility between and across organizations. However, it is also clear from these interviews that service providers and commissioners need to engage directly with young people and their families to build the confidence and expertise necessary to successfully negotiate the many aspects of transition from adolescence to adulthood.

## Conclusions

While there is an emerging national UK policy commitment to integrated commissioning,^4^ our study suggests that this is not followed through in the current commissioning arrangements for transitional care within health or between health and social care for young people with long-term conditions. Lack of integrated commissioning, together with a lack of guidance on transition to support integrated commissioning, hinders the processes of transition. Several of those interviewed identified that the adoption of integrated commissioning may well improve the organization and delivery of care for young people with long-term conditions. However, it will be important to evaluate whether such new organizational arrangements lead to better outcomes in terms of improvements in social participation and employment and the potential for reduction in later healthcare costs. Adoption of integrated commissioning will require a relational approach across all organizations responsible for the commissioning and delivery of transitional care involving mutual acknowledgement of professional expertise, agreed shared common values, understanding of training needs across all organizations, and joint ownership between relevant stakeholders.

In the light of the recent publication of the National Institute for Health and Care Excellence guidance^4^ on transition from child to adult services for young people and the new care models programme across the UK,^30^ there is a window of opportunity to examine and evaluate the impact of integrated commissioning for transitional care. This could facilitate the development of effective practical and feasible solutions for both the commissioning and provision of transitional care.

## Authors’ note

The views expressed in this article are those of the authors and not necessarily those of the NHS, the NIHR or the Department of Health. The funder took no part in the collection, analysis or interpretation of the data, in the writing of the article nor in the decision to submit the article for publication.
